# 

**Published:** 1851-01

**Authors:** 


***A Partner Wanted.***—We call the attention of all those whom it may concern, to the following advertisement.—*Ed.*

Dr. G. McDonald, of Macon, Ga., proposes to take a partner in the dental profession. A gentleman highly skilled in his profession, especially in the various modes of doing plate work, can get a good situation by early application, accompanied with proper vouchers.

*Dec.* 24, 1850.

